# Structure, function, and pathology of protein *O-*glucosyltransferases

**DOI:** 10.1038/s41419-020-03314-y

**Published:** 2021-01-12

**Authors:** Muhammad Zubair Mehboob, Minglin Lang

**Affiliations:** 1 CAS Center for Excellence in Biotic Interactions, College of Life Science, University of Chinese Academy of Sciences, Beijing, 100049 China; 2 College of Life Science, Hebei Agricultural University, Baoding, 071001 China

**Keywords:** Glycosylation, Oncogenes

## Abstract

Protein *O*-glucosylation is a crucial form of *O*-glycosylation, which involves glucose (Glc) addition to a serine residue within a consensus sequence of epidermal growth factor epidermal growth factor (EGF)-like repeats found in several proteins, including Notch. Glc provides stability to EGF-like repeats, is required for S2 cleavage of Notch, and serves to regulate the trafficking of Notch, crumbs2, and Eyes shut proteins to the cell surface. Genetic and biochemical studies have shown a link between aberrant protein *O*-glucosylation and human diseases. The main players of protein *O-*glucosylation, protein *O*-glucosyltransferases (POGLUTs), use uridine diphosphate (UDP)-Glc as a substrate to modify EGF repeats and reside in the endoplasmic reticulum via C-terminal KDEL-like signals. In addition to *O*-glucosylation activity, POGLUTs can also perform protein *O*-xylosylation function, i.e., adding xylose (Xyl) from UDP-Xyl; however, both activities rely on residues of EGF repeats, active-site conformations of POGLUTs and sugar substrate concentrations in the ER. Impaired expression of POGLUTs has been associated with initiation and progression of human diseases such as limb-girdle muscular dystrophy, Dowling–Degos disease 4, acute myeloid leukemia, and hepatocytes and pancreatic dysfunction. POGLUTs have been found to alter the expression of cyclin-dependent kinase inhibitors (CDKIs), by affecting Notch or transforming growth factor-β1 signaling, and cause cell proliferation inhibition or induction depending on the particular cell types, which characterizes POGLUT’s cell-dependent dual role. Except for a few downstream elements, the precise mechanisms whereby aberrant protein *O*-glucosylation causes diseases are largely unknown, leaving behind many questions that need to be addressed. This systemic review comprehensively covers literature to understand the *O*-glucosyltransferases with a focus on POGLUT1 structure and function, and their role in health and diseases. Moreover, this study also raises unanswered issues for future research in cancer biology, cell communications, muscular diseases, etc.

## Facts

- *POGLUTs*, similar to their *Drosophila* homolog *rumi*, add *O*-Glc to serine residues within the consensus sequences of EGF-like repeats found in several proteins and these repeats are essential for regulating many cellular functions.
- POGLUTs have a CAP10 catalytic domain involved in transferring Glc from UDP-Glc and a KDEL-like terminal signal, necessary for their binding to the KDEL receptors and recycling to the endoplasmic reticulum.
- Despite the glucosyltransferase activity, POGLUTs can also act as xylosyltransferases, i.e., adding Xyl from UDP-Xyl onto a serine residue.
- POGLUTs use both Notch and TGF-β1 pathway to manipulate the expression of CDKIs and, in turn, function in a cell type-dependent manner. Their overexpression increases cell proliferation in some cells, while inhibiting cell growth in other cells.

## Open questions

- Why and how can POGLUT1 display dual functionality, and why is this role cell-type specific?
- Which structural regions are different among the POGLUTs, which urge the modification of distinct sites within an EGF repeat, and why are POGLUT2 and 3 unable to modify POGLUT1 target site?
- Apart from the Notch pathway, what are the other possible pathways targeted by these glucosyltransferases affecting cell proliferation?

## Introduction

**Fig. 1:**
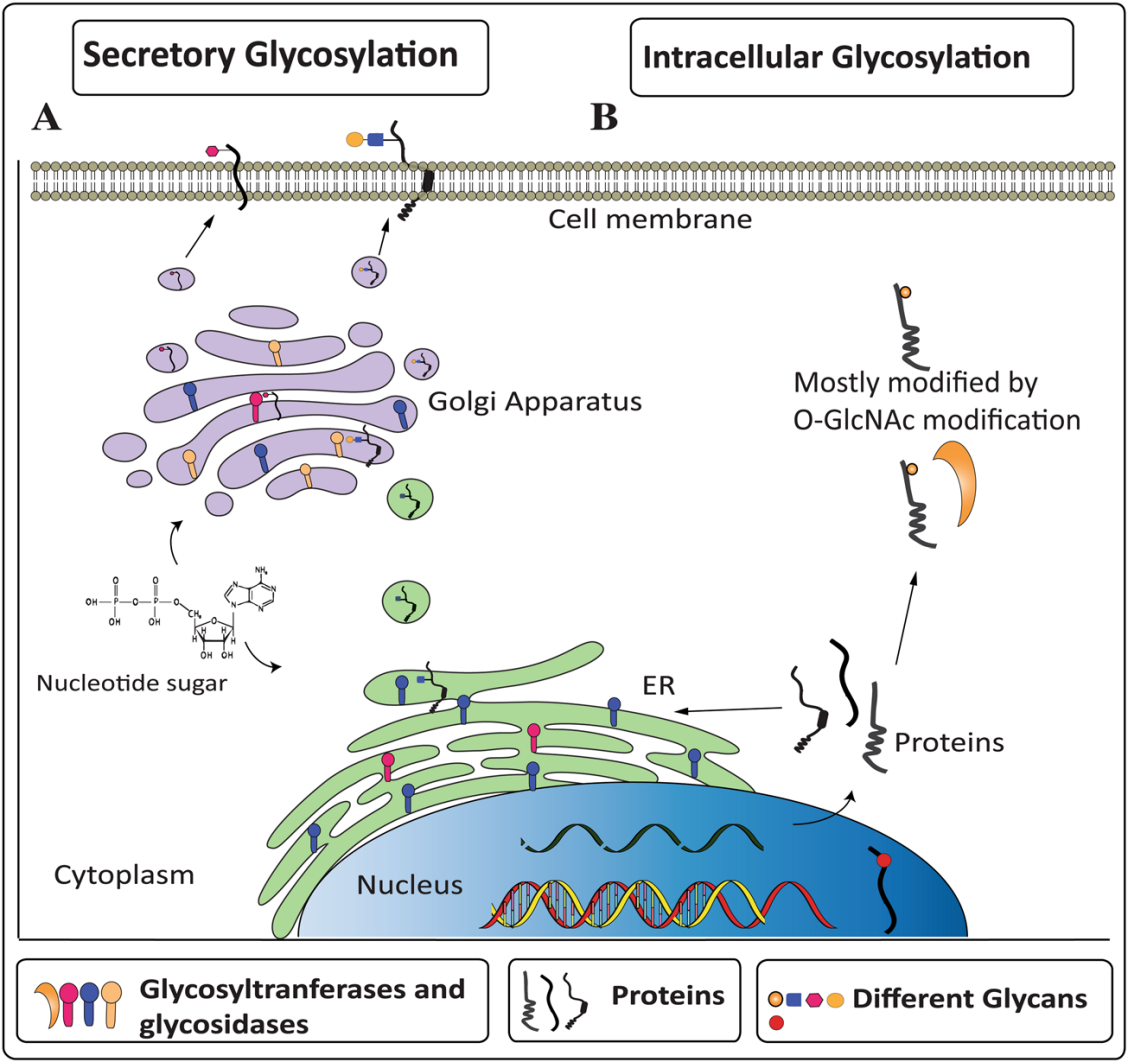
Cellular regulation and representation of glycans. Depending on the intracellular compartments, mechanisms altering the intracellular location of glycosyltransferases and glycosidases can influence and regulate glycans modfication. **A** Secretory glycosylation occurs mainly in the ER and Golgi apparatus for secreted or transmembrane proteins where glycosyltransferases add and extend glycan modification in an ordered sequential manner. Cellular receptors and secretory proteins are transported to the cell surface and extracellular space, respectively. **B** Intracellular glycosylation involves the addition of glycans by OGT to proteins that reside mainly in the cytoplasm and nucleus. This modification of a Ser/Thr residue has a short lifetime and is cleaved by OGA, providing a target site for the second same modification or phosphorylation of Ser/Thr.

Glycosylation, a non-templated and common enzymatic modification, involves the addition of glycans to macromolecules and is considered essential for cell viability and cellular responses in both uni- and multicellular organisms^1^. It adds structurally complex glycans assembled mainly by ten monosaccharides and occurs in different cell compartments, such as the cytoplasm and nucleus (intracellular glycosylation)^2^, and lumens of the endoplasmic reticulum (ER) and Golgi apparatus^3^ (secretory glycosylation; Fig. 1). For secretory glycosylation, one mammalian cell contains over 200 kinds of glycosyltransferases and glycosidases, whereas in the intracellular glycosylation, only *O-*linked *N*-acetylglucosamine transferase (OGT) and *N*-acetylglucosaminidase (OGA) are known to modify nuclear and cytosolic proteins^4^. Most of the translated proteins are primarily modified with *N*-linked glycans, but seven distinct types of *O-*linked glycans have also been found in humans, based on the first sugar binding to Ser/Thr residue^5^. Except for the cleavage of intact *N*-linked glycans by endoglycosidases and *O-*linked *N*-acetylglucosamine (*O-*GlcNAc) by OGA^4,6^, so far, no additional endoglycosidase for *O*-glycans has been reported, complicating the study of *O-*glycans in health and diseases. *O-*linked glycans are detected either on specific domains with consensus sequences, such as the epidermal growth factor (EGF)-like repeats and thrombospondin type 1 repeats (TSRs) or on collagen repeats present in collagen and adiponectin, in addition to some unknown set sequences such as the target site of OGT enzyme^5^. Apart from *O*-GlcNAc attachment to proteins in the cytosol and nucleus by OGT, protein modification by glycosyltransferases occurs in the ER and Golgi apparatus, where the addition of sugars takes place progressively.

**Fig. 2:**
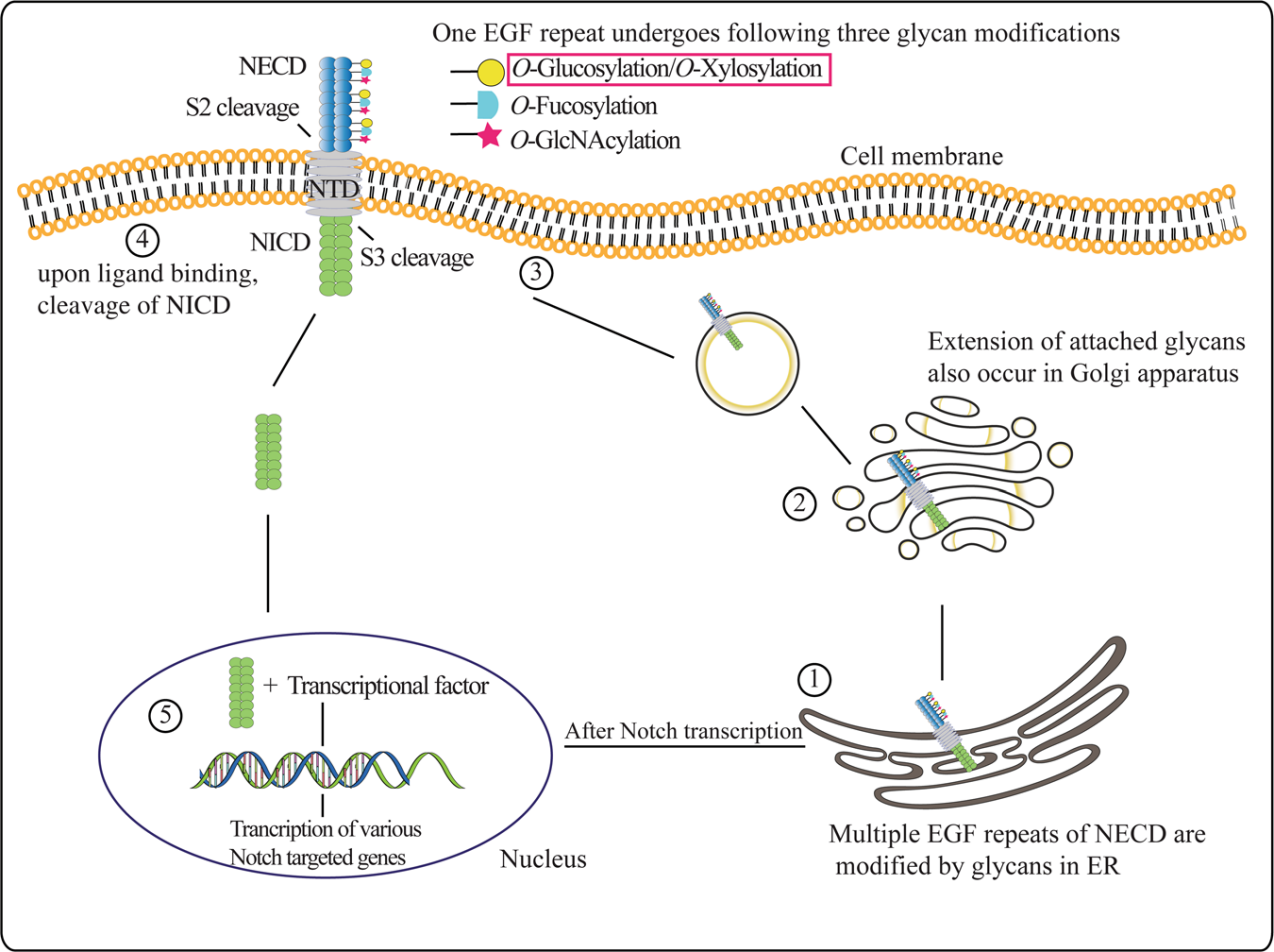
A representation of Notch structure, modification, and signaling. Notch receptor involved in several biological processes has three domains: NECD, NTD, and NICD. The NECD comprises a set of EGF-like repeats which undergo glycosylation. (1) The properly folded EGF repeats of NECD are modified with glycans added by glycan-specific enzymes and (2) these glycans are further extended in the Golgi apparatus or even also in the ER. (3) Sugar decorated Notch is transported to the cell membrane through membrane-bound vesicles. (4) After ligand (Delta, Jagged) binding, the ADAM proteases and γ-secretases execute Notch S2 and S3 cleavage, respectively, ultimately releasing the NICD that moves to the nucleus (5) where it binds the Notch pathway effector proteins to induce the expression of Notch target genes.

*O*-glucosylation refers to the addition of glucose (Glc) to EGF-like repeats of diverse substrates, such as coagulation factors VII, IX and protein Z^7^, Notch receptor and its ligands^8^, crumbs2^9^, Eyes shut (Eys) of *Drosophila*^10^, etc., some of which have shown impaired function after an aberrant glucosylation occurred. The biological importance of *O*-glucosylation has been highlighted in several cellular processes such as promoting mouse gastrulation by modifying the crumbs2 protein^9^, separating *Drosophila* rhabdomere by acting on Eys protein^10^, and playing a critical role in skin pigmentation and embryonic development through Notch signaling^11,12^. In humans, three protein *O-*glucosyltransferases (POGLUT1, 2, and 3) perform *O*-glucosylation activity by using uridine diphosphate (UDP)-Glc as a substrate^8,13^ and their homolog called Rumi performs equivalent function in *Drosophila*^14^. Alternatively, POGLUT1, 2, and 3 have been named as KTELC1, KDELC2, and KDELC3, respectively. These soluble enzymes accept only properly folded EGF-like repeats by rejecting linear peptides, suggesting that POGLUTs have a role in quality control of EGF-like repeats^15^. A single EGF-like repeat is a small protein motif with 30–40 AA (frequently repeated) and can vary in numbers from one in coagulation factors to >300 in *Drosophila* Dumpy protein. These repeats are found to be evolutionarily well conserved in the Notch receptor family and can be modified by *O-*fucosylation^16,17^, *O-*xylosylation^18^, and *O-N*-acetylglucosamine^19^, some of which play an important role in animal development^20^. There is only one Notch receptor in *Drosophila*, whereas humans have four Notch receptors. Both Notch1 and 2 possess 36 EGF repeats compared to Notch3 and 4 with 34 and 29 EGF repeats, respectively. Notch receptors harbor three domains named Notch extracellular domain (NECD), transmembrane domain, and intracellular domain (NICD), and for signaling to occur, ligand binding induces a proteolytic cleavage in the NECD, releasing the NICD to promote the transcription of target genes^21^ (Fig. 2). As the NECD is composed of multiple EGF repeats, posttranslational modifications of repeats may affect ligand binding and Notch cleavage. As Notch pathway conserved from *Drosophila* to human involves regulating cell fate during animal development, embryonic neurogenesis, cell proliferation, etc.^21–24^, impaired Notch signaling/glycosylation has been implicated in causing human pathologies, such as cerebrovascular disorder, cancer, leukoencephalopathy, etc.^25–30^.

Mutations in *POGLUT1* have been reported to cause human diseases showing phenotypical symptoms, such as Dowling–Degos disease 4 (DDD4) and limb-girdle muscular dystrophy-21 (LGMDR21), and are involved in reducing *O-*glucosylation activity on Notch^31,32^, thereby impairing Notch signaling in muscle and skin development. Furthermore, immunohistochemical staining for POGLUT1 is 50% weaker in DDD4 patients lesional skin compared to controls^12^, indicating its low expression in DDD4. It is worth noting that elimination of glucosyltransferases has a dual cellular impact on the cell surface presentation of Notch, which may depend on specific cell type or system. For instance, *rumi* mutant clones of *Drosophila* accumulated Notch both intracellularly and on the cell surface^14^, whereas deletion of *POGLUT1* in human embryonic kidney 293 (HEK293T) cells decreased plasma membrane expression of Notch1^33^. A recent effort to structurally elaborate the molecular mechanism of POGLUT1, 2, and 3 has tremendously highlighted the key points of *O*-glucosylation^34^; however, there is no comprehensive review written by considering their sequence-based structural features and functionality. Concerning the cell-specific dynamic role of POGLUT1, this systematic review focuses on structural domains and dual function of POGLUT1 along with all possible pathways experimentally observed in specific cell types. It also covers a wide range of literature along with suggestions to highlight the causative role of POGLUTs in human diseases.

### Sequence-based structural domains

**Fig. 3:**
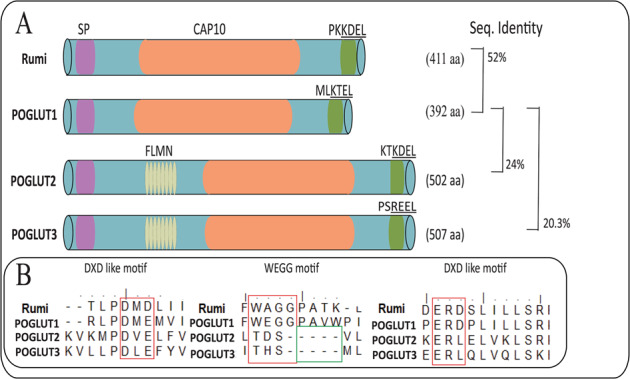
A comparison of structural domains of POGLUTs including Rumi. **A** *O*-glucosyltransferases have a signal peptide (SP) at the beginning of sequence, a CAP10 domain, and a ER-retention signal. Both KTEL and REEL retention signals have a lower affinity for ERD receptors than the KDEL signal. POGLUT1 shares 52% identity with Rumi and is the only enzyme that can rescue *O-*glucosylation function in *rumi* mutant flies. Although POGLUT2 and 3 show *O*-glucosyltransferase activity, but both enzymes are unable to rescue Rumi loss-of-function phenotype and only share 24% and 20.3% identity with POGLUT1, respectively, indicating different target sequence for modification. **B** Multiple sequence alignment of glucosyltransferases indicates the presence of DXD-like motifs, whose mutation can abolish *O*-glucosylation activity of enzymes. Moreover, POGLUT 2/3 lacks the WEGG motif considered necessary for UDP-glucose binding.

*POGLUTs*/*rumi* encoding glucosyltransferases possess a catalytic CAP10 domain and a KDEL-like localization signal. The CAP10 protein was first discovered in a *Cryptococcus neoformans* fungus^35^ and most proteins with CAP10 domain also have an ER-retention signal. BLAST analysis has indicated that POGLUT1 shares a high degree of identity (52%) with Rumi than POGLUT2 (37%) and POGLUT3 (38%), but the latter two proteins have an additional filamin-like domain (Fig. 3a). Out of FLAG-tagged POGLUT1, 2, and 3 constructs introduced into *rumi* mutant flies, only POGLUT1 could rescue the Rumi loss-of-function phenotype, although all three human POGLUTs were highly expressed in flies^18^. Recently, it has been demonstrated that POGLUT2/3 can add *O*-Glc to a serine residue within the consensus sequence C^3^-X-N-T-X-G-**S**-F-X-C^4^ of Notch1 EGF11 and Notch3 EGF10, a site distinct from the consensus sequence C^1^-X-**S**-X-P/A-C^2^ modified by POGLUT1/Rumi^13,36^. Hereby, both enzymes are unable to rescue the POGLUT1/Rumi loss-of-function phenotypes.

**Fig. 4:**
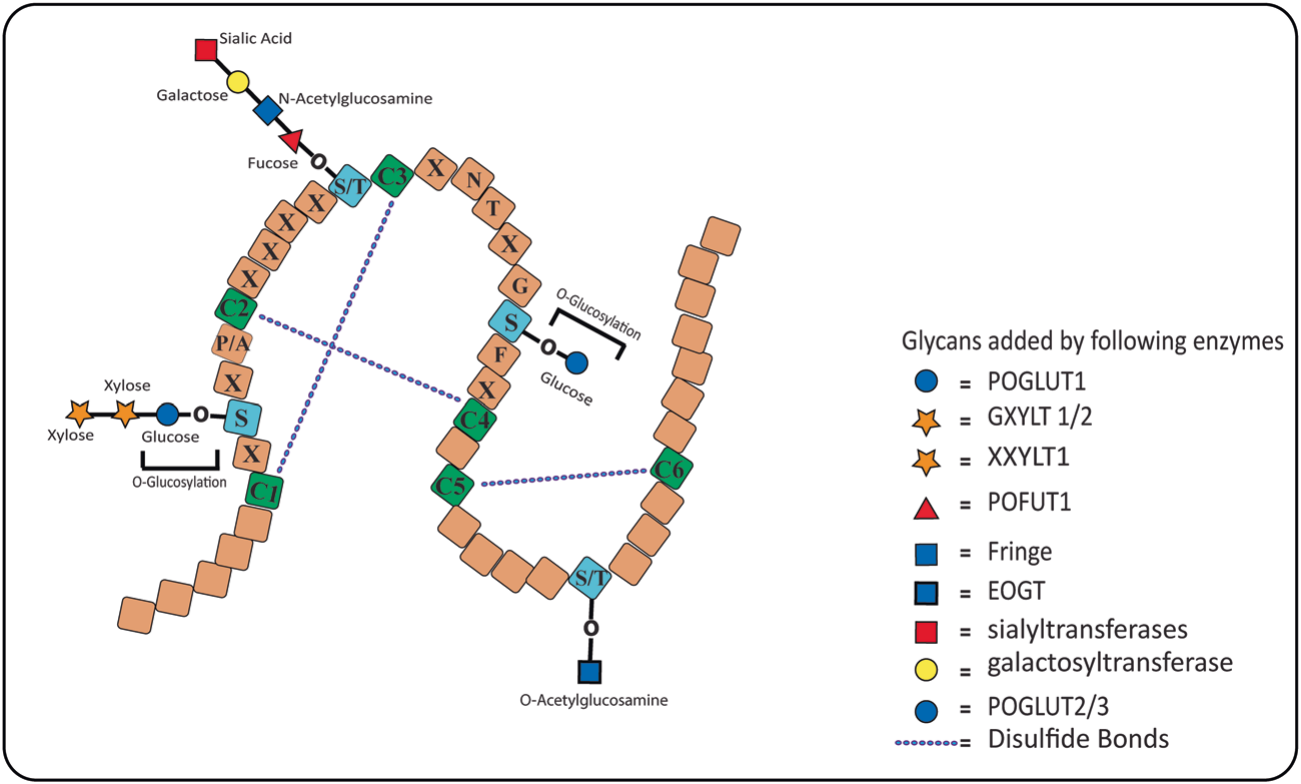
EGF-like repeat in the NECD domain and its modifications. Multiple EGF-like repeats present in the NECD harbor consensus sequences having a target site for glycosyltransferases. POGLUT1 adds *O*-Glc to a Ser residue in the consensus sequence C^1^-X-S-X-P/A-C^2^, which can further be extended to Xyl-Xyl-Glc-O trisaccharide by GXYLT1/2 and XXYLT1. The serine between C^1^ and C^2^ is highly conserved relative to other glycosylated sites, where both Ser and Thr can be found, and its the conformation of C^1^–C^2^ motif because of which POGLUTs can not glucosylate the Thr residue. POGLUT2 and 3 glucosylate a Ser residue conserved in C^3^–C^4^ of the EGF-like repeats and its further extension is not yet reported. POFUT1 adds *O-*Fuc to Ser/Thr residues between C^2^ and C^3^ of EGF repeats, which can further be elongated by Fringe enzyme. Moreover, the Ser/Thr residues between C^5^ and C^6^ is *O-*GlcNAcylated by a EOGT in *Drosophila* and mammals.

POGLUT1 encompasses two domains: an N-terminal domain having residues 1–180 and a C-terminal domain comprising residues 182–392, and both domains collectively form a UDP-Glc substrate-binding pocket involved in transferring Glc to EGF-like repeats. These repeats are characterized by six cysteine residues that are adequately spaced to form three disulfide bonds between C^1^–C^3^, C^2^–C^4^, and C^5^–C^6^ ^37^, and undergo specific posttranslational modifications by several glycosyltransferases (Fig. 4). Protein *O-*fucosyltransferase 1 (POFUT1) adds *O*-fucose (Fuc) to Ser/Thr residues within the C^2^–C^3^ (C^2^X_4-5_(**S/T**)C^3^) of EGF repeats^38–40^, which is further extended by a Fringe enzyme required for Notch-ligand binding^17,41^. An *O-*GlcNAc modification is also added to Ser/Thr residues located between C^5^ and C^6^ (C^5^-X-X-G-X-(**T/S**)-G-X-X-C^6^) by EGF domain-specific *O-*GlcNAc-transferase (EOGT) in *Drosophila*^19,42–44^ and mammals^45,46^, and contributes to epithelial cell–matrix interactions in *Drosophila*^43^, together with a role in human development^47^. POGLUTs and Rumi, unlike other glycosyltransferases that can modify both serine and threonine residues, only glucosylate serine residues in the C^1^C^2^ motif and structural studies of POGLUT1 provide an explanation that POGLUT1 possesses a catalytic base Asp133 that activates the hydroxyl group of serine, not threonine, by following an S_N_2 inversion mechanism^34,48^. When replacing serine, threonine fails to adopt the optimal posture for nucleophilic activation due to steric hindrance^48^, underlying the preference for serine over threonine for *O*-glucosylation.

*O*-Glc attached by POGLUT1, but not by POGLUT2/3, is extended to a di- or trisaccharide by adding xylose (Xyl); the first Xyl is mediated by α1-3xylosyltransferases (GXYLT1 and GXYLT2) and the second Xyl is added to Xyl-Glc-O disaccharide by α1-3Xyl-xylosyltransferase-1 (XXYLT1)^49,50^. Surprisingly, the first Xyl extension of *O-*Glc negatively regulates Notch trafficking to the cell membrane^51–53^ and destabilizes EGF repeats that can be restabilized by the addition of second Xyl^33^. Furthermore, Xyl extension also decreases the binding of Notch to and its activation by *trans*-ligand, but not *cis*-ligand, in *Drosophila*^54^. These findings explain the role of di-Xyl extension in EGF repeats stability and Notch trafficking.

### The catalytic CAP10 domain

Several critical residues of CAP10 domain directly interact with an EGF repeat to facilitate its access to the substrate-binding cleft formed by N- and C-terminal domains of POGLUTs^48^. Catalytic CAP10 domain of POGLUT1/Rumi has one WXGG and two DXD-like motifs (containing DME and ERD residues), which are essential for enzymatic activity. Studying POGLUT1’ interaction with hF7EGF1, hEGF12, and synthetic EGF(+), Li et al.^48^ has explained five hydrogen bonds and three regions of the EGF repeats: C^1^C^2^ loop (glucosylated motif), C^2^C^3^ loop (fucosylation motif), and a patch formed by C^4^(-2), C^6^(-1), and C^6^(-2) AA, which provide their accurate positioning for enzyme catalysis. Additional interactions are also formed by two POGLUT1 loops: loop 1 (238–242 AA) that folds over the C^1^C^2^ glucosylated motif and loop 2 (170–181 AA) that interacts with the UDP-Glc and C^2^C^3^ fucosylated motif. The C^1^C^2^ and C^2^C^3^ loop interactions constitute almost 80% of the EGF repeats surface buried in POGLUT1, whereas the remaining 20% is constituted by patch residues. In the glucosylated motif (C^1^X_a_**S**X_b_P/AC^2^), residues X_b_ and C^2^ form three hydrogen bonds with Gln240 of loop 1 and Ala172 of loop 2 in the POGLUT1–EGF complex, whereas the hydroxyl group of the serine residue forms the fourth hydrogen bond with Asp133, the POGLUT1 catalytic base^48^. As the asparagine and serine sidechains at X_b_ position make a strong hydrogen bond with Gln240, POGLUT1 prefers to interact with these two residues than arginine, which partially makes EGF repeats a poor substrate for POGLUT1 as observed in the case of mouse Notch1 EGF27, in which mutation of arginine to asparagine resulted in enhanced POGLUT1 activity^15^. The fifth hydrogen bond is developed between the NH group of C^2^(+2) residue in C^2^C^3^ loop and the carbonyl oxygen of POGLUT1 residue Pro178. In addition, notable are the extra interactions made by POGLUT1 Trp174 that is sandwiched between Pro175 and residues of C^2^C^3^ motif.

The chemical nature of “X” residues on either side of the serine (C^1^X_a_**S**X_b_P/AC^2^) can influence the efficiency of *O*-glucosylation by POGLUT1 as kinetic radioactive assays revealed that positively charged histidine and negatively charged aspartate at X_b_ position in mouse Notch1 EGF27 and Notch2 EGF16 decreased POGLUT1 activity^15^. The proline of C^1^C^2^ motif stabilizes the POGLUT1–EGF conformation required to generate hydrogen bonds, so residues bulkier than proline cannot be tolerated at this position. Proline and disulfide bonds at its subsites −2 and +3 enable the C^1^C^2^ motif (C^1^X_a_SX_b_P/AC^2^) to fold into a U-shaped loop, allowing the serine to penetrate deep into the POGLUT1 cleft. Even a single shift in serine position prevents its *O*-Glc modification, explaining the significance of serine position^55^. This also suggests that POGLUT1 modifies properly folded EGF repeats by developing multiple bonds. Moreover, the five-membered C^2^C^3^ motif (POFUT1 target) of hEGF domains also acts as a determinant for POGLUT1 to recognize specific target sequence^48^ and variability in motif length can make EGF repeats a poor substrate for POGLUT1 and POFUT1. The patch residue C^4^(-2) is closely associated with the conserved proline in the C^1^C^2^ motif and together these two residues make interactions with POGLUT1 residues Met103, Phe104, and Pro105. The latter two residues also develop interactions with variable residues C^6^(-1) and C^6^(-2) of the EGF repeats. Although similar in POGLUT1 and Rumi, POGLUT2/3 lacks these interacting residues, proposing their reason for targeting distinct site within EGF repeats.

Multiple sequence alignment of POGLUT1 with Rumi, POGLUT2, and 3 has disclosed the conservation of DXD-like motif in glucosyltransferases and lack of WXGG in the latter two (Fig. 3b). Previously, conserved tryptophan within the WXGG motif was found to be important in assisting substrate modification by creating interaction between the enzyme and UDP-Glc donors^56^. Meanwhile, mutation of the WX**G**G motif (G169E) in mouse POGLUT1 and equivalent G189E substitution in Rumi were detected to diminish enzyme activity^11,14^, pointing out an essential role of motif in Glc binding and substrate modification. In addition, the last glycine of WXGG motif (167–170 AA of POGLUT1) lies in loop 2 (170–181) of POGLUT1, of which residues Pro171, Ala172, Try174, and Pro178 assist in the successful transfer of Glc. Although POGLUT2 and 3 have displayed an ability to bind and transfer Glc^13^, but they lack a WXGG motif and other indispensable residues of POGLUT1 loop 2.

The classical metal-coordinating DXD motif is mainly shared by those glycosyltransferases that use nucleoside-diphosphate sugars and may contribute to the catalytic activity of enzymes as a systematic replacement of aspartate with alanine (DDD to ADD) in the Fringe DXD motif was found to abolish the Fringe activity completely^57^. POGLUTs and Rumi are independent of divalent ions for glucosylation activity; thus, DXD-like motifs may involve the interaction of *O*-Glc with divalent calcium ions bound to the short 5–7 AA spacer between each EGF repeat in the NECD^58^. The Arg218 (conserved in glucosyltransferases) in the second DXD-like motif E**R**D (217–219) of POGLUT1 and Arg279 coordinate with diphosphate of sugar to stabilize a negatively charged enzyme–substrate complex during the transition state of catalysis^48^. Moreover, expression of mutated Rumi (ERD (236–238) mutated to ERA) in *Drosophila* partially rescued *rumi* mutant phenotypes, whereas the ARA substitution of ERD showed too weak phenotypic rescue or complete inactivity^59^, reflecting the indeed requirement of ERD motif for *O*-glucosylation.

### The ER-retention function of KDEL-like signals

Protein retention signal is one of two ways for targeting soluble proteins to the ER and KDEL (Lys-Asp-Glu-Leu) is the preferred signal in higher vertebrates, but variants of KDEL motif such as prosite motifs ([KRHQSA]-[DENQ]-E-L)^60^ are also known to keep proteins ER resident. These motifs may also assist proteins to perform their functions. Compared with Rumi and POGLUT2 having a classical KDEL signal, POGLUT1 and 3 have the KTEL (Lys-Thr-Glu-Leu) and REEL (Arg-Glu-Glu-Leu) motifs, respectively. By interacting with three KDEL receptors (ERD21, 22, and 23) commonly located in the Golgi complex, soluble proteins with functional KDEL-like motif are retrieved from the Golgi back to the ER lumen via retrograde transport^61^. KDEL receptors show the characteristic pH-dependent binding for KDEL-like motifs as they have shown strong binding in the Golgi at pH 6 and become inactive in the ER at pH 7, resulting in release of proteins^62^. By normalizing the BiFC fluorescence signal for KDEL as 100%, KTEL motif showed a slightly weak binding signal upto 80% for KDEL receptors, whereas REEL exhibited 50% signal for ERD22 and 23, inferring different receptor-binding affinity of retention motifs^63^. Moreover, distinct chemical properties of residues in the KDEL-like motifs can also affect their receptor-binding affinity. These localization signals may also play a particular role in enzyme activity as observed for anterior gradient homolog 2, an enzyme involves promoting cell growth/metastasis, which lost the function when changing its KTEL signal to KDEL^64^. Acar et al.^14^ and Takeuchi et al.^18^ have explained a significant role of KDEL and KTEL for successful ER-retention of glucosyltransferases, but no study has examined in detail the relationship between the retention signals and glucosylation activity; therefore, the role of KDEL-like signals in POGLUT activities must be explored.

The chemical nature of -5 and -6 AA adjacent to retention signal can also influence the efficient ER retention of proteins, as various constructs of ERp18 motif (**HL**EDEL), usually having leucine and histidine at positions -5 and -6, respectively, revealed successful ER retention for constructs (**H**LEDEL or **H**AEDEL) comprising histidine or other positively charged residues, such as lysine (**K**LEDEL) at -6 position^65^. These constructs achieved considerable interactions with ERD receptors compared to secreted constructs (AAEDEL or EDEL) with other residues at the same place. Another construct with leucine at -5 position (A**L**EDEL) developed weak interactions with ERD22 and 23 and was found prominently in ER-Golgi co-localization^65^, indicating the significance of -5 position for receptor interaction. The presence of leucine at -5 position and the lack of histidine/positively charged residue at -6 of the POGLUT1 motif (**ML**KTEL) partially explains its low binding affinity for KDEL receptors and efficient ER retention. Taken together, it explains that *O-*glucosyltransferases with different localization signal motifs may have a different binding affinity for receptors and further experimental studies are needed to unearth the significant role of these motifs in enzymatic activities.

### Glucosyltransferase and xylosyltransferase activity

To examine the sugar specificity of mouse POGLUT1 against a variety of sugars using the EGF repeats of human factor VII (hFVII) as an acceptor substrate, it was established that POGLUT1 catalyzes the transfer of sugars from both UDP-Xyl and UDP-Glc^18^. Subsequent studies have produced conclusive evidence of Xyl and Glc utilization by other glucosyltransferases as well. Except for the methoxy group of Glc, both UDP sugars have the same orientation of hydroxyl groups, which facilitate their suitability into the same cleft^18^. Those motifs bearing diserine in consensus sequence (C^1^-X-S-S-P/A-C^2^) are considered most likely to be a target for *O-*xylosylation. Because in diserine motif (^50^CASSPC^55^) of hFVII EGF, a complete loss of *O-*glucosylation, as well as *O*-xylosylation modification in the S52A mutant and a reduction in *O*-xylosylation activity towards the S53A mutant were detected compared to wild-type motif^18^. Interestingly, an hFIX EGF repeat (^51^CESNPC^56^; lacking diserine) was also *O-*glucosylated and *O-*xylosylated, but it was a poor *O-*Xyl acceptor, similar to S53A mutant^18^. Using hEGF12 and EGF(+) substrates lacking diserine, it was experimentally demonstrated that both EGFs were xylosylated, but there was a twofold increase in *V*_max_ and an approximately twofold decrease in *K*_m_ for a hEGF12 mutant (C^1^VSSPC^2^) having diserine compared to the wild-type target^55^. Thus, unlike *O*-glucosylation activity, *O*-xylosylation is sensitive to the adjacent residues surrounding the S and second serine within the O-Glc consensus sequence enhances the efficacy of *O-*xylosylation^18^. Moreover, two local conformational states of POGLUT1 can likely be responsible for its ability to transfer both Glc and Xyl^55^. In UDP-CH2-Glc-based conformation, the C6-hydroxyl group of Glc simultaneously denotes a hydrogen bond to a β-phosphate oxygen atom and accepts a hydrogen bond from POGLUT1 residue Phe278 to increase the saccharide subsite volume for accommodating the Glc. In the second UDP-containing complex, POGLUT1 binds with UDP-Xyl and allows the C5 (without hydroxyl methyl group) of Xyl to develop similar interactions with Phe278, aligning sugar for S_N_2 attack^55^. Recently, POGLUT2 and 3 have been demonstrated to perform *O-*xylosylation function on Notch1 EGF11, a domain without diserine residues, but with low efficiency compared to glucosylation^13^. Besides, the relative catalytic efficiencies of both sugars may also depend on the available concentration of both sugars and the physiological conditions in the ER.

### Importance of *O*-glucosylation

Depending on the modification site within an EGF-like repeat, *O-*glucosylation can regulate Notch trafficking and cleavage, the liberation of NECD, Notch-ligand interactions, and Notch expression at the cell surface. *O-*glucosylation essentially provides stability to EGF-like repeats and, together with *O-*fucosylation, it is crucial for full Notch activity^66^. *O*-glucosylated sites in *Drosophila* Notch contribute to regulate its signaling especially at high temperature and act as a buffer against temperature-dependent loss of Notch signaling^67^. *O-*Glc modification between C^1^ and C^2^ of the EGF motif is critical to regulate Notch cleavage and cell surface expression. As in *rumi* mutant flies, upregulation and accumulation of cell surface Notch were observed without any interruption of Notch-Delta binding; however, Notch signaling pathway was defective due to impaired S2 cleavage, implying the importance of *O*-glucosylation in Notch cleavage^14^. Likewise, another study with *Poglut1*^−/−^ mouse and *Poglut1*-silenced C2C12 cells corroborated that *O*-Glc modification does not affect Notch-ligand binding, but can disrupt Notch activity at a step between the ligand binding and S3 cleavage^11^. *O*-Glc modification generally conforms to the NECD to expose the S2 cleavage site upon ligand binding. Moreover, reduced cell surface expression of Notch1 in *POGLUT1* knockout (KO) HEK293T cells, and *POGLUT1* and *POFUT1* double KO cells indicate the importance of *O*-Glc for cell surface expression of Notch^33^. Being at the binding interface between DLL4 ligand and Notch1 receptor, *O*-Glc modification of S^435^ between the C^3^ and C^4^ in human Notch1 EGF11 regulates ligand-induced Notch activation as Takeuchi et al.^13^ confirmed that *O-*Glu on the S^435^ position modulates cell surface presentation and DLL1-based activation of Notch1. They found that S435A mutation alone did not show a reduction in Notch activity, but a double mutation of S435A and *O*-Fuc position on EGF8, as well as on EGF12 caused a reduction of DLL1-based Notch1 activation^13^. The S435A and EGF8 *O*-Fuc double mutation disrupted cell surface presentation of Notch1, while both EGF12 *O*-Fuc and S435A mutations modulated the activity of DLL1 to activate Notch1. *O-*glucosylation, therefore, confers conformational stability to the NECD to enhance S2 cleavage and can govern ligand-dependent Notch activation if occurs on serine residues between C^3^ and C^4^ of the EGF repeats. Furthermore, the recognition of properly folded EGF repeats also explains the quality control function of *O*-glucosylation enzymes.

### Causative role of POGLUTs in human pathologies

The association between POGLUTs and human pathologies has been documented in many reports, and their link to the following diseases has been researched to date.

### Recessive LGMDR21

**Fig. 5:**
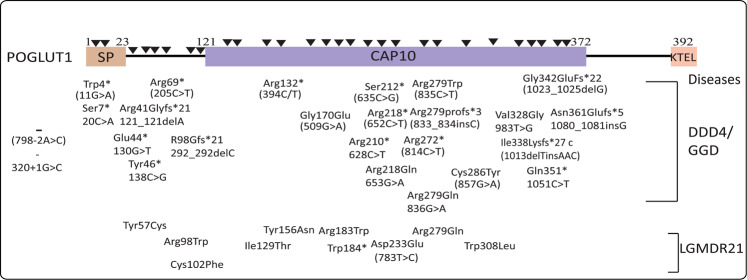
POGLUT1 mutations associated to cause human diseases. Several mutations of POGLUT1 have been implicated in human DDD4/GGD and LGMDR21. So far, only two nonsense mutations are reported in a signal peptide of POGLUT1 in DDD4/GGD patients. Most of the missense, nonsense, and frameshift variants of POGLUT1 are reported in the CAP10 catalytic domain, which harbors many essential amino acids required for *O*-glucosylation activity. A single change of essential residues of CAP10 domain can abolish *O*-glucosyltransferase activity as previously observed. The nonsense and frameshift mutations are resulted either in mRNA decay or the formation of a truncated POGLUT1 leading to DDD4/GGD and LGMDR21 disease.

Muscular dystrophy (MD), a group of disorders comprising 30 different kinds of muscular diseases, is a form of inherited disease characterized by constant breakdown and weakness of skeletal muscles over time. Continuous muscle damage is usually repaired by muscle**-**specific stem cells (SCs)^68^, whose pool is continuously regenerated and maintained in healthy muscles. Notch signaling is one of the main pathways for SCs regeneration, regulation, and quiescence^69,70^. So far, no primary SCs and Notch defects have been reported in MD; however, mutations of *POGLUT1* are implicated in causing LGMDR21. Such mutations generally reduce the enzymatic activity of POGLUT1, resulting in impaired Notch signaling and defective SCs regeneration including proliferation, differentiation, and depletion of SCs pool. For example, a POGLUT1 mutation D233E impaired O-Glc modification on Notch1 and decreased expression of NICD and HES-1, resulting in reduced muscle volume and self-renewal capacity of SCs in LGMDR21 patients than that in controls^71^. Several other mutations (Fig. 5) have also been described in *POGLUT1*, a few of which decrease its expression and others destabilize it. Immunofluorescence staining and western blotting of muscle biopsies of LGMDR21 patients revealed a reduction of the glycosylated form of α-dystroglycan (hypoglycosylation), Notch1 intracellular domain, and PAX7+ cells (dominant form of SCs in muscles)^31^. This study also described that I129T, R98W, C102F, and W308L mutations destabilized the POGLUT1, whereas R183W and Y57C mutations decreased the enzymatic activity as compared with control^31,72^. In summary, these findings uncover the underlying Notch-dependent pathogenic mechanism of POGLUT1 to cause muscular disorder and explain the importance of *O*-glucosylation in muscle development.

### Dowling–Degos and Galli–Galli disease

Besides its role in developmental processes, Notch signaling also plays a vital role in skin homeostasis and regulates the proliferation and differentiation of keratinocytes and melanocytes together with their interactions with each other^73,74^. Notch-induced abnormal pigmentation of skin has been previously described in the literature; for instance, the ablation of a transcription factor RBP-J (necessary for Notch signaling) in mice resulted in reduced melanoblast numbers within hair follicles, showing irregular pigmentation in the dermal papilla^75^. DDD4 is an autosomal dominant disorder characterized by reticular pigmentation, and was initially explained by Dowling and Freudenthal^76^. Both Galli–Galli (being a variant of DDD4) and DDD4 are characterized by the presence of hyperpigmented macules and papules on the chest, abdomen, trunk, and face. POGLUT1 shows a strong correlation with papules’ formation as immunohistologic staining for POGLUT1 was weaker in DDD4 patients than healthy controls^12^. Although the molecular mechanism of disease is yet unknown, a study of 13 unrelated DDD4 individuals, including 5 patients previously characterized by Hanneken^77^, determined six mutations (Trp4*, Arg69*, Arg132*, Ser212*, Arg279Trp, and Gly342Glufs*22) in *POGLUT1* by whole-exome sequencing, which were involved to cause DDD4^12^. Later, ~17 novel frameshifts, nonsense, and missense mutations were reported^32,78–80^, which are represented in Fig. 5. The frameshift and nonsense mutations are likely to result in either truncated protein or nonsense-mediated mRNA decay, while missense mutations reduce POGLUT1 expression or activity. For example, exon trapping analysis of frameshift c.320 + 1 G > C mutation in *POGLUT1* revealed a complete skipping of exon 3, resulting in disruption of the functionally important POGLUT1 N-terminal domain^32^. These authors also evaluated the effect of missense mutations on POGLUT1 activity and examined that, unlike Val328Gly mutant protein that was not detected in culture media and degraded as an unstable form, the secretion level of Arg279Gln mutant was similar to wild-type POGLUT1, but it was enzymatically dysfunctional^32^. As aforementioned, both Arg218 and Arg279 of POGLUT1 are involved in substrate binding, so substitution of these residues may likely decrease POGLUT1 activity and substrate affinity. Moreover, small interfering RNA (siRNA)-mediated knockdown of POGLUT1 in melanocyte-derived cell lineage MZ7-MEL altered mRNA expression of Notch signaling components and enhanced the expression of *MITF* (microphthalmia-associated transcription factor) and *TYR* (gene of tyrosinase). MITF is essential for melanocytes development^81^ and regulates the expression of TYR^82^ needed to convert tyrosine to melanin. Taken together, this data reflect the combined effect of Notch signaling, MITF, and TYR to misdirect melanin deposition leading to hyperpigmented macules across the entire body.

### Acute myeloid leukemia and acute lymphoblastic leukemia

Association between abnormal proliferation of blood leukocytes and aberrant *O*-glucosylation has been demonstrated in recent years^83^, but the underlying mechanism is not yet clear. The first report of POGLUT1’ association with acute myeloid leukemia (AML) was by Teng et al.^84^ when it was isolated and detected in human AML cells transformed from myelodysplastic syndrome CD34^+^ cells. Among 12 normal human tissues, high level of *POGLUT1* mRNA in both the liver and spleen (organs for normal and extramedullary hematopoiesis) suggest that POGLUT1 seems to participate in the development of blood cells^84^. Subsequently, a higher growth rate of POGLUT1 overexpressed U937 cells (monocytic leukemia cell line) in comparison to cells without exogenous POGLUT1 had confirmed the association of POGLUT1 with AML progression, which might occur by inhibiting the CDKIs through Notch signaling^84^.

Inactivation of p15 and p16 has been observed in AML and acute lymphoblastic leukemia^85^; thus, POGLUT1-associated AML was initially considered p15 or p16 dependent. Even though, this perception was immediately declined when p15 and p16 expression level did not change in POGLUT1-downregulated U937 cells showing proliferation inhibition^86^. As HES-1, a transcription factor in Notch signaling induces cell proliferation by p27 inhibition^87,88^, another study demonstrated that downregulation of POGLUT1 in U937 cells inhibited cell proliferation by decreasing HES-1 and accumulated CDKI p27 to cease cell division^83^. HES-1 is also required to repress p21 and p57 in many tissues^89,90^; hence, the speculation of cell proliferation by these CDKIs may also be considered. Overall, the basic route of leukemia cell proliferation induced by POGLUT1 is not fully unveiled and should be addressed extensively in future studies.

### Hepatic and pancreatic dysfunction

Being an ER-resident protein, POGLUTs expression can also relate to the ER stress response. Hepatocytes and pancreatic β-cells have extensive ability to synthesize and export proteins, so ER stress can alter genes expression in these cells. Interestingly, ELISA assay of patients with hepatic dysfunction reported upregulation of POGLUT2 compared to that in healthy controls^91^. Accordingly, ER stress by tunicamycin in hepatoblastoma HepG2 cells also increased POGLUT2 expression level that induced the cell cycle arrest^91^, suggesting an association of POGLUT2 with hepatic diseases. Several studies have also confirmed an association between ER stress and β-cell dysfunction or type 2 diabetes. Upon exposing β-cell line INS-1 to palmitate, high expression of POGLUT2 appeared, which in turn activated caspase-3-dependent apoptosis^92^. In contrast, POGLUT2 inhibition in INS-1 cells reduced expression of apoptotic factor caspase-3 along with attenuation of ER stress^92^, implying a link between POGLUT2 and β-cell apoptosis. Future research is needed to explore the role of POGLUT2 in regulating the physiological process of hepatocytes and pancreatic cells.

Also, POGLUT1 mutation has been reported in primary biliary cholangitis (PBC), a chronic and progressive autoimmune liver disease induced by disruption of small bile ducts. A single-nucleotide polymorphism (rs2293370: susceptible C-allele) identified at position 3q13.33 through a genome-wide association study increased the expression of *POGLUT1*^93^, which might be involved in the pathogenesis of PBC, possibly by inducing the Notch pathway, as Notch signaling is involved in regulating the development of peripheral immune responses^94^.

### Cell-dependent dual role of POGLUT1 in inhibiting and promoting cell proliferation

Presently, the dual function of POGLUT1 affecting cell proliferation has been discussed, which is cell-type specific and very similar to cell-dependent dual function of Notch. For example, overactivation of Notch in small-cell lung cancer (SCLC) cells and endothelial cells has been shown to promote p21- and p27-dependent cell cycle arrest^95,96^, whereas the oncogenic role of Notch is identified in lymphoblastic T-cell leukemia and fibroblasts that leads to cancer development^97,98^. Both down- and upregulation of POGLUT1 have been noticed to alter the expression level of CDKIs by affecting Notch or transforming growth factor-β1 (TGF-β1) pathway and cause cell proliferation inhibition and induction.

**Fig. 6:**
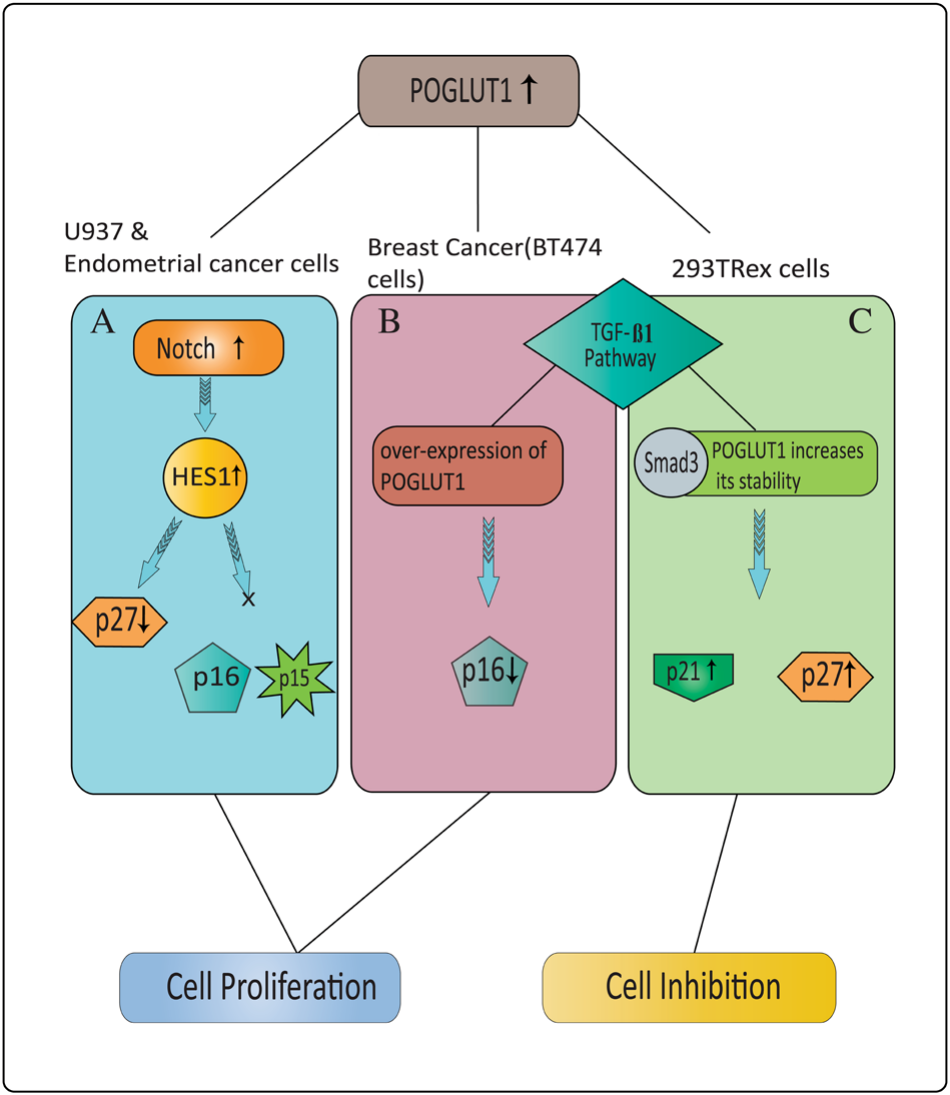
Partial signaling pathways reported based on specific cell types by POGLUT1 for cell proliferation and inhibition. Dual role of POGLUT1 in cell cycle progression or supression is cell-type specific. Up to now, a complete signaling pathway for cell proliferation or inhibition in response to overexpression of POGLUT1 is not fully explained; however, POGLUT1’ association with Notch and TGF-β1 signaling pathways has been observed. **A** In U937 and endometrial cancer cells, higher expression of POGLUT1 increases Notch signaling and HES-1 level, which in turn decreases P27 level without altering p15 and p16, and induces cell cycle progression or tumorigenesis. **B** Evidently, decreased expression level of p16 upon overexpressing POGLUT1 has also been observed, which explains the involvement of more than one signaling route in regulating CDKIs level. Upregulated POGLUT1 reduces Smad3 phosphorylation in breast cancer cells by repressing p16 expression in the presence of TGF-β1, thus promoting BT474 cell proliferation. **C** In contrast, overexpression of POGLUT1 in 293TRex cells triggers cell cycle arrest and increases Smad3 protein stability via inhibiting its proteasomal degradation. In fact, POGLUT1 enhances TGF-β1 signaling by modulating Smad3 expression and increases level of CDKIs p27 and p21, which bind with CDKs enzymes to block cell proliferation.

By reducing the expression of POGLUT1, exogenous miR-134 overexpression in human endometrial SCs (HuECSCs) downregulated Notch signaling proteins Notch1 and HES-1, and markedly enhanced p27 concentration to repress HuECSCs mutiplication, thereby elucidating the inhibitory role of POGLUT1 in endometrial cancer cells^99^ (Fig. 6a, reverse in the figure). Similarly, downregulated *POGLUT1* in colorectal cancer (CRC) cells that showed significantly higher expression of POGLUT1 than noncancerous cells prevented illegitimate CRC cell proliferation^100^. Further, immunostaining analysis of mouse POGLUT1 in mouse (C57BL6/J model) lungs described its higher expression in alveolar and bronchiolar epithelia along with its two- to threefold elevation in non-SCLC (NSCLC), explaining the association of POGLUT1 with lung cancer^101^. Consistently, genetic silencing of *POGLUT1* in A549 and H23 NSCLC cells caused cell cycle arrest by decreasing the transcript level of Notch effectors HEY1 and HES2^101^. In contrast, the role of POGLUT1 to increase 293TRex cell proliferation has also been documented, as the downregulation of POGLUT1 by siRNA in 293TRex cells moderately increased cell proliferation companied with a decrease in p21 and p27 level^102^, explaining that POGLUT1 could also regulate cell cycle progression.

TGF-β1 is the second pathway to be affected by POGLUT1. Several communications have studied the significance of TGF-β1 in CDKIs induction and cell cycle inhibition^103^. TGF-β1 signaling regulates cellular processes by phosphorylation of SMAD proteins, which bind with coSMAD to act as a transcriptional factor and participate in regulating target gene expression. It was observed in human breast cancer cells BT474 that POGLUT1 overexpression in the presence of TGF-β1 improved cell viability and inhibited p16 upregulation through TGF-β1 pathway, suggesting the involvement of POGLUT1 in BT474 cell proliferation^104^ (Fig. 6b). As TGF-β1 signaling pathway induces p16 expression through phosphorylated Smad3, exogenous POGLUT1 overexpression in BT474 cells reduced the endogenous phosphorylation of Smad3^104^, leading to TGF-β1 dependent role of POGLUT1 in the BT474 cell cycle. Contrarily, Smad3 also showed high expression and stability in response to POGLUT1 overexpression in 293TRex cells, in which POGLUT1 increased p21 and p27 expression through TGF-β1 and contributed to Smad3 stability via inhibiting its ubiquitin-proteasomal degradation, making the cells susceptible to TGF-β1 dependent cell cycle arrest^105^ (Fig. 6c). The mechanism of Notch-induced modulation of TGF-β1 has also been discussed which can be considered in future studies^106,107^. Together, these findings reflect the partially observed pathways used by POGLUT1 for dual functionality, but additional efforts are needed to find the cell-specific role of POGLUT1.

## Conclusion and future perspective

Research into the glycobiology with help of modern genetics and genome engineering technologies has contributed to underscore the important regulatory roles of sugar modifications. As discussed above, the responsible enzymes for *O*-glucosylation, POGLUTs, modify the consensus sequences within EGF repeats of Notch by adding Glc, which involves the structural and functional characterization of the Notch. Further work in area of Notch glycobiology can be expected by considering the following questions; how can *O*-Glc change the conformation of NECD and stabilize the EGF repeats? What is the role of *O*-Glc in S2 cleavage of Notch activation? Furthermore, it is also necessary to find the relationships of POGLUTs with other human diseases and what are the pathomechanisms behind them. Therefore, it takes a lot of scientific work to ascertain the role of protein *O*-glucosylation and its exact relationship with human diseases.

## References

[1] Reily, C., Stewart, T. J., Renfrow, M. B. & Novak, J. Glycosylation in health and disease. *Nat. Rev. Neurol*. **15**, 346–366 (2019). 10.1038/s41581-019-0129-4 PMC6590709 30858582

[2] Zachara, N., Akimoto, Y. & Hart, G. W. in *Essentials of Glycobiology*. 3rd edn (Cold Sping Harbor, NY, 2017).

[3] Stanley P (2011). Golgi glycosylation. Cold Spring Harb. Perspect. Biol..

[4] Hart, G. W. & Akimoto, Y. in *Essentials of Glycobiology*. 2nd edn (Cold Sping Harbor, NY, 2009).

[5] Moremen KW, Tiemeyer M, Nairn AV (2012). Vertebrate protein glycosylation: diversity, synthesis and function. Nat. Rev. Mol. Cell Biol..

[6] O’Donnell N, Zachara NE, Hart GW, Marth JD (2004). Ogt-dependent X-chromosome-linked protein glycosylation is a requisite modification in somatic cell function and embryo viability. Mol. Cell Biol..

[7] Hase S (1988). A new trisaccharide sugar chain linked to a serine residue in bovine blood coagulation factors VII and IX. J. Biochem..

[8] Shao L, Luo Y, Moloney DJ, Haltiwanger RS (2002). O-glycosylation of EGF repeats: identification and initial characterization of a UDP-glucose: protein O-glucosyltransferase. Glycobiology.

[9] Ramkumar N (2015). Protein O-glucosyltransferase 1 (POGLUT1) promotes mouse gastrulation through modification of the apical polarity protein CRUMBS2. PLoS Genet..

[10] Haltom AR (2014). The protein O-glucosyltransferase Rumi modifies eyes shut to promote rhabdomere separation in Drosophila. PLoS Genet..

[11] Fernandez-Valdivia R (2011). Regulation of mammalian Notch signaling and embryonic development by the protein O-glucosyltransferase Rumi. Development.

[12] Basmanav FB (2014). Mutations in POGLUT1, encoding protein O-glucosyltransferase 1, cause autosomal-dominant Dowling-Degos disease. Am. J. Hum. Genet..

[13] Takeuchi H (2018). Two novel protein O-glucosyltransferases that modify sites distinct from POGLUT1 and affect Notch trafficking and signaling. Proc. Natl Acad. Sci. USA.

[14] Acar M (2008). Rumi is a CAP10 domain glycosyltransferase that modifies Notch and is required for Notch signaling. Cell.

[15] Takeuchi H, Kantharia J, Sethi MK, Bakker H, Haltiwanger RS (2012). Site-specific O-glucosylation of the epidermal growth factor-like (EGF) repeats of notch: efficiency of glycosylation is affected by proper folding and amino acid sequence of individual EGF repeats. J. Biol. Chem..

[16] Moloney DJ (2000). Mammalian Notch1 is modified with two unusual forms of O-linked glycosylation found on epidermal growth factor-like modules. J. Biol. Chem..

[17] Moloney DJ (2000). Fringe is a glycosyltransferase that modifies Notch. Nature.

[18] Takeuchi H (2011). Rumi functions as both a protein O-glucosyltransferase and a protein O-xylosyltransferase. Proc. Natl Acad. Sci. USA.

[19] Matsuura A (2008). O-linked N-acetylglucosamine is present on the extracellular domain of notch receptors. J. Biol. Chem..

[20] Haltom AR, Jafar-Nejad H (2015). The multiple roles of epidermal growth factor repeat O-glycans in animal development. Glycobiology.

[21] Kopan R, Ilagan MXG (2009). The canonical Notch signaling pathway: unfolding the activation mechanism. Cell.

[22] Aster JC (2014). In brief: Notch signalling in health and disease. J. Pathol..

[23] Fiúza U-M, Arias AM (2007). Cell and molecular biology of Notch. J. Endocrinol..

[24] Fortini ME (2009). Notch signaling: the core pathway and its posttranslational regulation. Dev. Cell.

[25] Li X (2009). Notch3 signaling promotes the development of pulmonary arterial hypertension. Nat. Med..

[26] Bolós V, Grego-Bessa J, de la Pompa JL (2007). Notch signaling in development and cancer. Endocr. Rev..

[27] Stanley P, Okajima T (2010). Roles of glycosylation in Notch signaling. Curr. Top. Dev. Biol..

[28] Takeuchi H, Haltiwanger RS (2010). Role of glycosylation of Notch in development. Semin. Cell Dev. Biol..

[29] Urata Y, Takeuchi H (2020). Effects of Notch glycosylation on health and diseases. Dev. Growth Differ..

[30] Bianchi S, Dotti MT, Federico A (2006). Physiology and pathology of notch signalling system. J. Cell Physiol..

[31] Servián-Morilla, E., et al. POGLUT1 biallelic mutations cause myopathy with reduced satellite cells, α-dystroglycan hypoglycosylation and a distinctive radiological pattern. *Acta Neuropathol*. **139**, 565–582 (2020). 10.1007/s00401-019-02117-6 PMC7196238 31897643

[32] Ralser DJ (2018). Altered Notch signaling in Dowling-Degos disease: additional mutations in POGLUT1 and further insights into disease pathogenesis. J. Investig. Dermatol..

[33] Takeuchi H (2017). O-Glycosylation modulates the stability of epidermal growth factor-like repeats and thereby regulates Notch trafficking. J. Biol. Chem..

[34] Yu H, Takeuchi H (2019). Protein O-glucosylation: another essential role of glucose in biology. Curr. Opin. Struct. Biol..

[35] Chang Y, Kwon-Chung K (1999). Isolation, characterization, and localization of a capsule-associated gene, CAP10, of *Cryptococcus neoformans*. J. Bacteriol..

[36] Ogawa M (2019). A new type of *O*-glucose modification by POGLUT2 and POGLUT3 controls Notch signaling and trafficking. Trends Glycosci. Glycotechnol..

[37] Chang J-Y, Li L, Lai H (2001). A major kinetic trap for the oxidative folding of human epidermal growth factor. J. Biol. Chem..

[38] Luo Y, Haltiwanger RS (2005). O-fucosylation of notch occurs in the endoplasmic reticulum. J. Biol. Chem..

[39] Okajima T, Reddy B, Matsuda T, Irvine KD (2008). Contributions of chaperone and glycosyltransferase activities of O-fucosyltransferase 1 to Notch signaling. BMC Biol..

[40] Wang Y (2001). Modification of epidermal growth factor-like repeats with O-fucose molecular cloning and expression of a novel gdp-fucose protein O-fucosyltransferase. J. Biol. Chem..

[41] Brückner K, Perez L, Clausen H, Cohen S (2000). Glycosyltransferase activity of Fringe modulates Notch–Delta interactions. Nature.

[42] Müller R, Jenny A, Stanley P (2013). The EGF repeat-specific O-GlcNAc-transferase Eogt interacts with notch signaling and pyrimidine metabolism pathways in *Drosophila*. PLoS ONE.

[43] Sakaidani Y (2011). O-Linked-N-acetylglucosamine on extracellular protein domains mediates epithelial cell–matrix interactions. Nat. Commun..

[44] Ogawa M, Senoo Y, Ikeda K, Takeuchi H, Okajima T (2018). Structural divergence in O-GlcNAc glycans displayed on epidermal growth factor-like repeats of mammalian notch1. Molecules.

[45] Sakaidani Y (2012). O-linked-N-acetylglucosamine modification of mammalian Notch receptors by an atypical O-GlcNAc transferase Eogt1. Biochem. Biophys. Res. Commun..

[46] Sawaguchi S (2017). O-GlcNAc on NOTCH1 EGF repeats regulates ligand-induced Notch signaling and vascular development in mammals. Elife.

[47] Shaheen R (2013). Mutations in EOGT confirm the genetic heterogeneity of autosomal-recessive Adams-Oliver syndrome. Am. J. Hum. Genet..

[48] Li Z (2017). Structural basis of Notch O-glucosylation and O-xylosylation by mammalian protein–O-glucosyltransferase 1 (POGLUT1). Nat. Commun..

[49] Whitworth GE, Zandberg WF, Clark T, Vocadlo DJ (2009). Mammalian Notch is modified by D-Xyl-α1-3-D-Xyl-α1-3-D-Glc-β1-O-Ser: implementation of a method to study O-glucosylation. Glycobiology.

[50] Sethi MK (2012). Molecular cloning of a xylosyltransferase that transfers the second xylose to O-glucosylated epidermal growth factor repeats of notch. J. Biol. Chem..

[51] Lee TV (2013). Negative regulation of Notch signaling by xylose. PLoS Genet..

[52] Urata Y (2020). Xylosyl extension of O-glucose glycans on the extracellular domain of NOTCH1 and NOTCH2 regulates Notch cell surface trafficking. Cells.

[53] Matsumoto K (2016). Dual roles of O-glucose glycans redundant with monosaccharide O-fucose on Notch in Notch trafficking. J. Biol. Chem..

[54] Lee TV, Pandey A, Jafar-Nejad H (2017). Xylosylation of the Notch receptor preserves the balance between its activation by trans-Delta and inhibition by cis-ligands in *Drosophila*. PLoS Genet..

[55] Yu H (2016). Structural analysis of Notch-regulating Rumi reveals basis for pathogenic mutations. Nat. Chem. Biol..

[56] Busch C, Hofmann F, Gerhard R, Aktories K (2000). Involvement of a conserved tryptophan residue in the UDP-glucose binding of large clostridial cytotoxin glycosyltransferases. J. Biol. Chem..

[57] Munro S, Freeman M (2000). The Notch signalling regulator fringe acts in the Golgi apparatus and requires the glycosyltransferase signature motif DXD. Curr. Biol..

[58] Hambleton S (2004). Structural and functional properties of the human Notch-1 ligand binding region. Structure.

[59] Lee TV, Takeuchi H, Jafar-Nejad H (2010). Regulation of Notch signaling via O-glucosylation: Insights from *Drosophila* studies. Methods Enzymol..

[60] Hulo N (2006). The PROSITE database. Nucleic Acids Res..

[61] Stornaiuolo M (2003). KDEL and KKXX retrieval signals appended to the same reporter protein determine different trafficking between endoplasmic reticulum, intermediate compartment, and Golgi complex. Mol. Biol. Cell.

[62] Bräuer P (2019). Structural basis for pH-dependent retrieval of ER proteins from the Golgi by the KDEL receptor. Science.

[63] Raykhel I (2007). A molecular specificity code for the three mammalian KDEL receptors. J. Cell Biol..

[64] Gupta A, Dong A, Lowe AW (2012). AGR2 gene function requires a unique endoplasmic reticulum localization motif. J. Biol. Chem..

[65] Alanen HI, Raykhel IB, Luukas MJ, Salo KE, Ruddock LW (2011). Beyond KDEL: the role of positions 5 and 6 in determining ER localization. J. Mol. Biol..

[66] Takeuchi H, Haltiwanger RS (2014). Significance of glycosylation in Notch signaling. Biochem. Biophys. Res. Commun..

[67] Leonardi J, Fernandez-Valdivia R, Li Y-D, Simcox AA, Jafar-Nejad H (2011). Multiple O-glucosylation sites on Notch function as a buffer against temperature-dependent loss of signaling. Development.

[68] Collins CA (2005). Stem cell function, self-renewal, and behavioral heterogeneity of cells from the adult muscle satellite cell niche. Cell.

[69] Mourikis P, Tajbakhsh S (2014). Distinct contextual roles for Notch signalling in skeletal muscle stem cells. BMC Dev. Biol..

[70] Bjornson CR (2012). Notch signaling is necessary to maintain quiescence in adult muscle stem cells. Stem Cells.

[71] Servián‐Morilla E (2016). A POGLUT1 mutation causes a muscular dystrophy with reduced Notch signaling and satellite cell loss. EMBO Mol. Med..

[72] Ross J (2012). Defects in glycosylation impair satellite stem cell function and niche composition in the muscles of the dystrophic Largemyd mouse. Stem Cells.

[73] Okuyama R, Tagami H, Aiba S (2008). Notch signaling: its role in epidermal homeostasis and in the pathogenesis of skin diseases. J. Dermatol. Sci..

[74] Moriyama M (2006). Notch signaling via Hes1 transcription factor maintains survival of melanoblasts and melanocyte stem cells. J. Cell Biol..

[75] Aubin-Houzelstein G (2008). Melanoblasts’ proper location and timed differentiation depend on Notch/RBP-J signaling in postnatal hair follicles. J. Invest. Dermatol..

[76] Dowling G, Freudenthal W (1938). Acanthosis nigricans. Proc. R. Soc. Med..

[77] Hanneken S (2011). Morbus Galli-Galli. Der Hautarzt.

[78] Duchatelet S (2018). A new nonsense mutation in the POGLUT 1 gene in two sisters with Dowling‐Degos disease. J. Eur. Acad. Dermatol. Venereol..

[79] Kono M (2019). A Japanese case of Galli-Galli disease due to a previously unreported POGLUT1 mutation. Acta Derm. Venereol..

[80] Wilson N (2017). Mutations in POGLUT 1 in Galli–Galli/Dowling–Degos disease. Br. J. Dermatol..

[81] Hsiao JJ, Fisher DE (2014). The roles of microphthalmia-associated transcription factor and pigmentation in melanoma. Arch. Biochem. Biophys..

[82] Yasumoto K-i, Yokoyama K, Shibata K, Tomita Y, Shibahara S (1994). Microphthalmia-associated transcription factor as a regulator for melanocyte-specific transcription of the human tyrosinase gene. Mol. Cell Biol..

[83] Ma W (2011). hCLP46 regulates U937 cell proliferation via Notch signaling pathway. Biochem. Biophys. Res. Commun..

[84] Teng Y (2006). Cloning, expression and characterization of a novel human CAP10-like gene hCLP46 from CD34+ stem/progenitor cells. Gene.

[85] Herman JG (1997). Distinct patterns of inactivation of p15INK4B and p16INK4A characterize the major types of hematological malignancies. Cancer Res..

[86] Wang Y (2010). Overexpression of human CAP10-like protein 46 KD in T-acute lymphoblastic leukemia and acute myelogenous leukemia. Genet. Test. Mol. Biomark..

[87] Murata K (2005). Hes1 directly controls cell proliferation through the transcriptional repression of p27Kip1. Mol. Cell Biol..

[88] Monahan P, Rybak S, Raetzman LT (2009). The Notch target gene HES1 regulates cell cycle inhibitor expression in the developing pituitary. Endocrinology.

[89] Castella P, Sawai S, Nakao K, Wagner JA, Caudy M (2000). HES-1 repression of differentiation and proliferation in PC12 cells: role for the helix 3-helix 4 domain in transcription repression. Mol. Cell Biol..

[90] Riccio O (2008). Loss of intestinal crypt progenitor cells owing to inactivation of both Notch1 and Notch2 is accompanied by derepression of CDK inhibitors p27Kip1 and p57Kip2. EMBO Rep..

[91] Wang X (2016). KDELC1, a novel endoplasmic reticulum resident glycoprotein in hepatic dysfunction. Int. J. Clin. Exp. Med..

[92] Zhang J (2018). Inhibiting KDELC1 may prevent palmitate-induced apoptosis in INS-1 cells by reducing ER stress. Int. J. Clin. Exp. Med..

[93] Hitomi Y (2019). POGLUT1, the putative effector gene driven by rs2293370 in primary biliary cholangitis susceptibility locus chromosome 3q13. 33. Sci. Rep..

[94] Radtke F, MacDonald HR, Tacchini-Cottier F (2013). Regulation of innate and adaptive immunity by Notch. Nat. Rev. Immunol..

[95] Sriuranpong V (2001). Notch signaling induces cell cycle arrest in small cell lung cancer cells. Cancer Res..

[96] Noseda M (2004). Notch activation induces endothelial cell cycle arrest and participates in contact inhibition: role of p21Cip1 repression. Mol. Cell Biol..

[97] Zlobin, A., Bloodworth, J. C., Baker, A. T. & Osipo, C. *Notch Signaling Pathway in Carcinogenesis* (Springer, 2019).

[98] Pancewicz J (2010). Notch signaling contributes to proliferation and tumor formation of human T-cell leukemia virus type 1–associated adult T-cell leukemia. Proc. Natl Acad. Sci. USA.

[99] Gao Y, Liu T, Huang Y (2015). MicroRNA‐134 suppresses endometrial cancer stem cells by targeting POGLUT1 and Notch pathway proteins. FEBS Lett..

[100] Fang H (2017). Human CAP10-like protein 46 kDa gene promotes malignancy in colorectal cancer. OMICS J. Integr. Biol..

[101] Chammaa M (2018). RUMI is a novel negative prognostic marker and therapeutic target in non–small‐cell lung cancer. J. Cell Physiol..

[102] Chu Q, Liu L, Wang W (2013). Overexpression of hCLP 46 enhances Notch activation and regulates cell proliferation in a cell type‐dependent manner. Cell Prolif..

[103] Gordon KJ, Blobe GC (2008). Role of transforming growth factor-β superfamily signaling pathways in human disease. Biochim. Biophys. Acta Mol. Basis Dis..

[104] Jin G (2014). Protein O‑glucosyltransferase 1 overexpression downregulates p16 in BT474 human breast cancer cells. Oncol. Lett..

[105] Xing Y (2015). hCLP46 increases Smad3 protein stability via inhibiting its ubiquitin-proteasomal degradation. Protein Cell.

[106] Masuda S (2005). Notch1 oncoprotein antagonizes TGF‐β/Smad‐mediated cell growth suppression via sequestration of coactivator p300. Cancer Sci..

[107] Sun Y (2005). Notch4 intracellular domain binding to Smad3 and inhibition of the TGF-β signaling. Oncogene.

